# Supplementary material for: Multi‐dataset identification of innovative feature genes and molecular mechanisms in keratoconus
Source: J Cell Mol Med. 2024 Sep 19;28(18):e70079. doi: 10.1111/jcmm.70079 (PMC11412914; doi:10.1111/jcmm.70079)
Supplement: Supplementary file 2 — Material S2. [file JCMM-28-e70079-s002.zip › 7.GSEA analysis/2.ATOH7/KEGG_PROTEASOME.html]

Details for gene set KEGG\_PROTEASOME[GSEA]

|  || Dataset | ATOH7.group.cls#h\_versus\_l |
| Phenotype | group.cls#h\_versus\_l |
| Upregulated in class | l |
| GeneSet | KEGG\_PROTEASOME |
| Enrichment Score (ES) | -0.3104711 |
| Normalized Enrichment Score (NES) | -1.1090245 |
| Nominal p-value | 0.2631579 |
| FDR q-value | 1.0 |
| FWER p-Value | 0.996 |
Table: GSEA Results Summary

  

Fig 1: Enrichment plot: KEGG\_PROTEASOME      
 Profile of the Running ES Score & Positions of GeneSet Members on the Rank Ordered List

  

| SYMBOL | TITLE | RANK IN GENE LIST | RANK METRIC SCORE | RUNNING ES | CORE ENRICHMENT || 1 | POMP | na | 10769 | 0.070 | -0.2705 | Yes |
| 2 | PSMA8 | na | 10785 | 0.069 | -0.2311 | Yes |
| 3 | PSMD12 | na | 11743 | 0.056 | -0.2266 | Yes |
| 4 | PSMC4 | na | 12260 | 0.049 | -0.2131 | Yes |
| 5 | PSMC3 | na | 12953 | 0.041 | -0.2095 | Yes |
| 6 | PSMA2 | na | 13392 | 0.036 | -0.2013 | Yes |
| 7 | PSME3 | na | 13656 | 0.034 | -0.1895 | Yes |
| 8 | PSMD2 | na | 13774 | 0.033 | -0.1740 | Yes |
| 9 | PSMB9 | na | 14109 | 0.029 | -0.1667 | Yes |
| 10 | PSMC6 | na | 14151 | 0.029 | -0.1513 | Yes |
| 11 | PSMF1 | na | 14154 | 0.029 | -0.1347 | Yes |
| 12 | PSMB6 | na | 14634 | 0.024 | -0.1345 | Yes |
| 13 | PSMB2 | na | 14638 | 0.024 | -0.1206 | Yes |
| 14 | PSMD11 | na | 14690 | 0.024 | -0.1084 | Yes |
| 15 | PSMD6 | na | 14721 | 0.023 | -0.0958 | Yes |
| 16 | PSMD14 | na | 14848 | 0.022 | -0.0865 | Yes |
| 17 | PSMD1 | na | 15133 | 0.020 | -0.0832 | Yes |
| 18 | PSMC1 | na | 15436 | 0.017 | -0.0820 | Yes |
| 19 | PSMD8 | na | 15596 | 0.016 | -0.0773 | Yes |
| 20 | PSMA6 | na | 15710 | 0.015 | -0.0719 | Yes |
| 21 | PSMB5 | na | 16237 | 0.011 | -0.0808 | Yes |
| 22 | PSMB1 | na | 16874 | 0.006 | -0.0959 | Yes |
| 23 | PSMA7 | na | 17328 | 0.002 | -0.1081 | Yes |
| 24 | PSMD4 | na | 17857 | -0.003 | -0.1217 | Yes |
| 25 | PSMD7 | na | 18001 | -0.004 | -0.1234 | Yes |
| 26 | PSMA5 | na | 18180 | -0.006 | -0.1254 | Yes |
| 27 | PSME2 | na | 19167 | -0.014 | -0.1456 | Yes |
| 28 | PSMB3 | na | 19200 | -0.015 | -0.1381 | Yes |
| 29 | PSMB7 | na | 19545 | -0.017 | -0.1379 | Yes |
| 30 | PSMA3 | na | 19581 | -0.018 | -0.1287 | Yes |
| 31 | PSMB4 | na | 19671 | -0.019 | -0.1205 | Yes |
| 32 | PSME1 | na | 20286 | -0.025 | -0.1240 | Yes |
| 33 | PSMC5 | na | 21938 | -0.042 | -0.1474 | Yes |
| 34 | PSMB8 | na | 22094 | -0.044 | -0.1266 | Yes |
| 35 | PSMD13 | na | 22306 | -0.046 | -0.1060 | Yes |
| 36 | IFNG | na | 24472 | -0.077 | -0.1244 | Yes |
| 37 | PSME4 | na | 24584 | -0.079 | -0.0825 | Yes |
| 38 | PSMA1 | na | 24673 | -0.080 | -0.0391 | Yes |
| 39 | PSMA4 | na | 24804 | -0.082 | 0.0044 | Yes |
| 40 | PSMB10 | na | 25512 | -0.095 | 0.0384 | Yes |
| 41 | PSMC2 | na | 25725 | -0.099 | 0.0889 | Yes |
| 42 | PSMD3 | na | 26926 | -0.124 | 0.1253 | Yes |
| 43 | PSMB11 | na | 28816 | -0.173 | 0.1704 | Yes |
Table: GSEA details [plain text format]

  

Fig 2: KEGG\_PROTEASOME      
 Blue-Pink O' Gram in the Space of the Analyzed GeneSet

  

Fig 3: KEGG\_PROTEASOME: Random ES distribution      
 Gene set null distribution of ES for **KEGG\_PROTEASOME**
